# The First High-Throughput Sequencing-Based Study of Viruses Infecting Solanaceous Crops in Kosovo Reveals Multiple Infections in Peppers by Six Plant Viruses

**DOI:** 10.3390/plants14091273

**Published:** 2025-04-22

**Authors:** Burim Ismajli, Zsuzsanna N. Galbács, András Péter Takács, Éva Várallyay

**Affiliations:** 1 Genomics Research Group, Department of Plant Pathology, Institute of Plant Protection, Hungarian University of Agriculture and Life Sciences, Szent-Gyorgyi Albert Street 4, H-2100 Gödöllő, Hungary; nagyne.galbacs.zsuzsanna@uni-mate.hu (Z.N.G.); varallyay.eva@uni-mate.hu (É.V.); 2 Department of Plant Protection, Institute of Plant Protection, Hungarian University of Agriculture and Life Sciences, Deák Ferenc Street 17, H-8360 Keszthely, Hungary; takacs.andras.peter@uni-mate.hu

**Keywords:** plant virus, solanaceous crops, virome, HTS, Balkan region

## Abstract

High-throughput sequencing (HTS) was employed for the first time to investigate plant viruses infecting solanaceous crops, including potato (*Solanum tuberosum*), tomato (*Solanum lycopersicum*), and pepper (*Capsicum annuum*), in Kosovo. Leaf samples showing virus-like symptoms were collected from various regions during the summer of 2023. Based on ribodepleted RNA sequencing and bioinformatics analysis, six viruses were identified: cucumber mosaic virus, broad bean wilt virus 2 (BBWV2), potato virus Y, pepper cryptic virus 2 (PCV2), bell pepper endornavirus (BPEV), and ranunculus white mottle virus. BBWV2, PCV2, and BPEV are reported for the first time in the Balkan region. Virus presence was validated using RT-PCR. Phylogenetic analyses revealed that the identified viral strains did not cluster according to their hosts and geographical origins. CMV and BBWV2 variants exhibited reassortment events, indicating possible local evolution or novel virus introductions. This research highlights the widespread occurrence of mixed infections in pepper plants and highlights the need for additional research into the virus transmission dynamics and potential reservoir hosts. These findings emphasize the need for continuous surveillance and integrated plant protection strategies to mitigate the impacts of viral infections on pepper and other economically important crops in Kosovo.

## 1. Introduction

Kosovo, officially known as the Republic of Kosovo, is located in the heart of Southeast Europe. It is a landlocked country bordered by Albania, Montenegro, Serbia, and North Macedonia. Covering an area of 10,887 km^2^ and home to approximately 1.7 million people, it boasts a diverse landscape of high plains, rolling hills, and towering mountains. With a predominantly continental climate influenced by Mediterranean and alpine features, Kosovo possesses rich cultural heritage and plays a significant role in the Balkan region, both culturally and historically.

Agricultural cultivation areas in the country have increased in recent years, reaching about 19,500 hectares in 2022. Kosovo’s vegetable production is a vital sector for both the economy and the livelihoods of local farmers. One of the most cultivated solanaceous crops is pepper. Its widespread use as a vegetable and spice has caused to it be cultivated worldwide. Pepper, grown in 16% of Kosovo’s cultivated fields, is a popular vegetable crop. The region’s favorable climate offers optimal growing conditions for its open-field cultivation. Kosovo’s pepper industry is a significant contributor to the country’s economy. The most common varieties include Somborka, Kurtovska Kapija, Feferoni (chili), Duga Bela, and Shorok Shari. The pepper processing industry in Kosovo has a long history, with over 10 large companies handling over 90% of the country’s total pepper production. Open-field pepper cultivation is usually achieved using seedlings; however, direct seed sowing is occasionally used, especially for the processing industry. High-quality seedling preparation involves using suitable substrates, maintaining optimal conditions (temperature, light, humidity, and nutrition), and ensuring that seeds are of high quality, are disinfected, and are preferably hybrid F1 varieties for reliability. Kosovo’s pepper production has seen significant growth, but the presence of plant viruses poses a threat to its yields and quality.

The viromes of tomato and pepper have been investigated for over two decades, including surveys in the Balkan region. Pepper diseases have been surveyed in a joint effort by five countries: Albania, Bulgaria, Greece, Macedonia, and Serbia [1]. Using serological and RT-PCR methods, cucumber mosaic virus (CMV), tobacco mosaic virus (TMV), potato virus Y (PVY), tomato mosaic virus (ToMV), and tomato spotted wilt virus (TSWV) were identified as the most frequent viruses. A subsequent ELISA screen of pepper growing in Macedonia revealed high infection rates for CMV, alfalfa mosaic virus (AMV), and PVY [2]. A RT-PCR-based survey of tomatoes in Serbia revealed that they were highly infected with CMV and PVY, while infections with AMV, TSWV, ToMV, and TMV were also present [3]. Interestingly, surveys performed in other parts of the world yielded similar results. A large RT-PCR-based survey of pepper in Korea showed that pepper was highly infected with BBWV2, with co-infection with CMV, pepper mottle virus (PMV), pepper mild mottle virus (PMMV), and PVY often observed [4].

Although ELISA and RT-PCR can detect the presence of any particular pathogen in the tested plant material, high-throughput sequencing (HTS) provides a comprehensive and unbiased analysis of the entire virome present in the sample, enabling the identification of known and previously undescribed viruses. This powerful tool can detect low-abundance viruses that may be missed using traditional diagnostic methods [5]. In Slovenia, an HTS-based survey of symptomatic and asymptomatic tomatoes and surrounding weeds revealed the presence of 125 viruses, of which 79 had not been described previously, revealing the unexpected complexity of the viruses present in rural samples [6]. The use of HTS for virome characterization in pepper revealed a more detailed picture of the viruses infecting this plant species. Specifically, PCV2 and BPEV were identified worldwide, including Slovakia, Panama, Korea, Slovenia, Tennessee (USA), and Poland, and in almost all of the tested pepper plants, suggesting their latent background and infectious nature [7,8,9,10,11,12].

CMV (*Cucumovirus*) belongs to the *Cucumovirus* genus within the *Bromoviridae* family and is recognized as one of the most prevalent and economically important plant viruses [13]. Its positive-sense RNA genome is segmented, encoding the viral replicase on RNA1 (3.3 kb) and RNA2 (3.0 kb), as well as the movement protein and the coat protein on RNA3 (2.2 kb). On RNA2, an additional protein, ORF2b, is expressed and acts as a viral silencing suppressor molecule [14]. CMV infects various crops, and more than 1200 species, representing more than 100 families, have been identified as its hosts so far. It is present around the world and is widespread in the Balkan region. Its presence has been reported in Macedonia, Bosnia and Herzegovina, Bulgaria, Greece, and Serbia [2,15,16,17] (Table S1). Its broad host range and genetic variability pose a persistent threat to agriculture, highlighting the importance of ongoing monitoring and research to mitigate its global impact [18]. The virus is primarily transmitted by a wide range of aphids in a non-persistent manner, but it can also spread through parasitic plant dodder and, in rare cases, via seeds [19]. CMV leads to significant yield losses and fruit deformities, along with consequent economic losses in crops, especially when coexisting with other viruses like TMV and PVY [1].

BBWV2 (*Fabavirus betaviciae*), belonging to the genus *Fabavirus* within the *Secoviridae* family, has a bipartite genome composed of two single-stranded positive-sense RNAs. Both RNA1 and RNA2 encode a single open reading frame (ORF) that is translated into a polyprotein precursor, which is subsequently cleaved into functional proteins. RNA-dependent RNA polymerase (RdRp) is encoded on RNA1, while the movement protein and two coat proteins are encoded on RNA2 [4,20]. BBWV2 is widely distributed worldwide and infects both monocotyledonous and dicotyledonous crops, including peppers [20,21]. BBWV2 is transmitted by aphids (especially by *Aphis gossypii* and *Myzus persicae*) in a non-persistent manner [22,23,24]. Symptoms of BBWV2 in peppers include necrotic spots on leaves and stems, stunted growth, and apical necrosis [22].

PVY (*Potyvirus yituberosi*) is a filamentous, non-enveloped virus from the *Potyvirus* genus within the *Potyviridae* family. It has a single-stranded, positive-sense RNA genome, encoding a large polyprotein and a smaller protein called PIPO. Distributed worldwide, PVY infects a broad range of hosts from nine families, but its most significant economic impact is seen predominantly within solanaceous crops [25,26]. It has also been reported and found to be widespread in the Balkans (Table S1). PVY is spread predominantly by vegetative propagation but can be transmitted through a non-persistent transmission mechanism by more than 40 aphid species, including *Myzus persicae* [25,27]. Different pathotypes of PVY can cause vein banding, vein necrosis, and interactions with resistance genes on pepper [28,29].

PCV2 (*Deltapartitivirus duocapsici*) is a bipartite double-stranded RNA virus within the genus *Deltapartitivirus* of the family *Partitiviridae*. Viruses belonging to this family are effectively transmitted through pollen and seeds but are not spread via grafting or mechanical inoculation, and they lack known natural vectors. The first descriptions of PCV2 came from Hungary, where it was identified in the Hungarian pepper cultivar “Hungarian Wax” based on its dsRNA sequences [30]. Since its original description, it has been found exclusively in pepper and chili but in different parts of the world, including India [31], China [32], and Tennessee, USA, where symptomatic and symptomless peppers were infected with this virus [11]. In Europe, it has been reported in Slovakia [7] and Poland [12], but its presence could not be directly correlated with any symptom.

BPEV (*Alphaendornavirus capsici*), classified within the family *Endornaviridae*, is a persistent double-stranded RNA virus known to infect pepper [33]. It has a genome of approximately 14.7 kb in length and is transmitted through seeds but not via graft inoculation. The BPEV genome comprises a single open reading frame (ORF) that encodes a large polyprotein with several conserved functional domains [33]. BPEV induces mild crinkling and chlorosis on young leaves and reduces fruit yields and quality in bell peppers [34], but its presence can also be completely latent [33]. BPEV has been found in Israel [35], Panama [8], Colombia [36], and the USA [11]. Although BPEV’s impact is evident in bell pepper cultivars, in which severe symptoms have been reported, hot pepper cultivars display mild symptoms [9]. In Europe, it has been detected in Slovakia and Poland [12,37]. Furthermore, sweet pepper plants infected with BPEV have been found in the Middle East, including Lebanon and Syria, marking the significant expansion of its known geographic distribution [38].

RWMV (*Ophiovirus ranunculi*) is a member of *Aspivirideae* in the *Ophiovirus* genus. It was first isolated from an ornamental *Ranunculus* hybrid in Liguria, Italy, in 1990, showing leaf mottle and distortion [39]. Since that time, it has been reported in *Anemone* [40], tomato, pepper, and *Solanum nigrum* grown in Slovenia [6,10], Australia [41], and Greece [42] (Table S1). Its single-stranded negative-sense RNA genome comprises at least three separately encapsidated RNA segments [43]. The full genome sequences of the four RNAs are also available from NCBI GenBank, sequenced by the DSMZ from a *Lactuca sativa* collected in Germany (OR879093-6). The virus can be mechanically transmitted to various herbaceous host plants, but no vector has been identified to date.

In this research, we investigated the viromes of symptomatic solanaceous crops, including potato, tomato, and pepper, from different locations in Kosovo using an HTS-based metagenomic approach. Although we tested the viromes of only 12 plants, infections in tomato with CMV and in pepper with six different viruses, almost exclusively as co-infections, were revealed. The validation of the HTS results with unbiased RT-PCR allowed us to reliably investigate the virus variants and their phylogenetic relationships in order to explain their presence in peppers in the Balkans.

## 2. Results

### 2.1. Determining the Viromes of Solanaceous Crops Grown in Kosovo Using HTS

In the summer of 2023, virus-like symptoms were observed in vegetable fields across Kosovo, prompting an investigation into plant viruses infecting potato, tomato, and pepper. HTS was applied as an advanced and unbiased technique for virome characterization. A single sequencing library was constructed to represent all of the sampled plant species (potatoes, tomatoes, and peppers) (Table S2). The sequencing yielded 21,558,420 raw reads, which were processed to generate 21,442,968 high-quality trimmed reads. The de novo assembly of these reads resulted in 40,362 contigs (Table S3). The BLAST (https://blast.ncbi.nlm.nih.gov/Blast.cgi (accessed on 28 February 2025)) analysis of these contigs revealed the presence of nine viruses (CMV, BBWV2, PVY, PCV2, BPEV, RWMV, TVCV, TAV, and CMV satellite) represented by at least one contig with significant homology (0 E-value) to the reference genomes of known plant-infecting viruses (Table S4). The contigs usually covered the whole genomes of the viruses, except in the case of TVCV, TAV, and CMV satellite. In these cases, the contigs were shorter than the viral or satellite genomes, and the other regions showed similarity to the solanaceous genomes, indicating their host genome origins. In the case of TVCV, the identity of the contigs to the reference genome was quite long, which could suggest the presence of host-integrated segments from a previous infection. Hits to the other six viruses appeared valid and were analyzed further using virus-specific RT-PCR.

### 2.2. Identification of CMV Infection in Tomato and Pepper Samples

**Figure 1:**
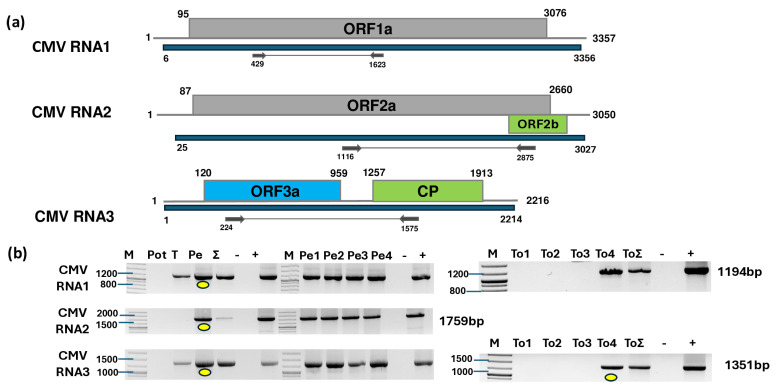
Results indicating the presence of CMV. (**a**) Schematic representation of the CMV genome and the positions of the primers used for RT-PCR validation. The blue line indicates the region covered by the HTS sequencing results. (**b**) RT-PCR results for virus validation. M represents the GeneRuler 100 bp Plus DNA Ladder; Pot indicates potato; T indicates tomato; Pe indicates the pepper pool; Σ denotes the combined pool of Pot, T, and Pe that was sequenced; −/+ stand for negative and positive controls, respectively; Pe1, Pe2, Pe3, and Pe4 denote individual pepper plants; and To1, To2, To3, and To4 denote individual tomato plants. Yellow dots indicate the products that were cloned and sequenced.

In the case of CMV, three, four, and nine contigs mapped separately to the three segments of the CMV genome (Figure 1a). In total, 387,085 reads mapped to RNA1, 319,933 reads mapped to RNA2, and 1,248,670 reads mapped to RNA3, covering 100% of the CMV genome (Table S4).

**Figure 2:**
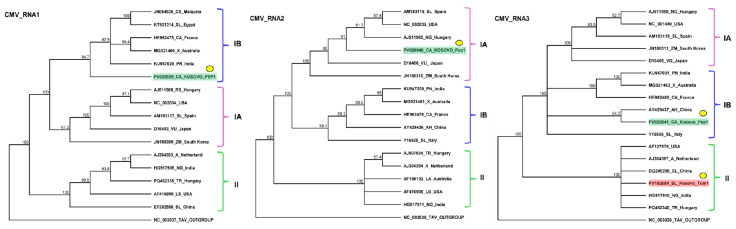
Phylogenetic analysis of the CMV variants. The analysis was conducted using Geneious Tree Builder with the Tamura–Nei model, the neighbor-joining method, and 1000 bootstrap replicates. Different colors indicate clusters of CMV subgroups. Green and red boxes highlight the CMV variants found in pepper and tomato, respectively. Yellow dots indicate the variants sequenced in this study. The sequences are indicated by their GenBank accession numbers. The middle panel of the legend indicates the host plant species using the following abbreviations: CA—*Capsicum annuum*, CS—*Cucumis sativus*, SL—*Solanum lycopersicum*, X—*Xanthosoma*, PN—*Piper nigrum*, RS—*Raphanus sativus*, VU—*Vigna unguiculata*, ZM—*Zea mays*, A—*Alstroemeria*, NG—*Nicotiana glutinosa*, TR—*Trifolium repens*, LS—*Lactuca saligna*, AH—*Arachis hypogaea*, and LA—*Lupinus angustifolius*.

To validate the presence of all three segments of CMV and inspect its presence in the different species and individuals, we designed primers based on the contig sequences and sequences of diverse CMV variants (Table S5). RT-PCR revealed the presence of CMV in the tomato and pepper pools. Only one tomato and all four pepper individuals were infected with the virus (Figure 1b). For tomato, we cloned and sequenced the PCR products of RNA3 using the cDNA prepared from the individuals as a template (GenBank accession number PV102684) (Table S6). For pepper, the PCR products originating from the cDNA prepared from the pepper pool served as the template. These products were subjected to Sanger sequencing and SNP analysis. As the PCR products showed no SNPs, we cloned and sequenced these products amplified from RNA1, RNA2, and RNA3 (GenBank accession number PV020939-41) and used them for phylogenetic analysis (Table S7). The sequencing of the presented CMV variants revealed that the tomato plant was infected with a variant belonging to the less virulent subgroup II of CMV (Figure 2).

The variant present in the pepper appeared to be a reassortant of subgroup I variants. While RNA1 and RNA3 clustered into subgroup IB, RNA2 clustered into subgroup IA. The RNA1 of the KOSPep variant was notably distinct from those of the other CMV variants in GenBank, with its closest homolog (HE962478—sequenced in France from a *Capsicum* sp.) showing 92.552% identity (Table S8). The closest homolog of KOSPep CMV RNA2 in GenBank was a variant sequenced in Spain from a tomato host (AM183118—60.356% identity) (Table S8), although they did not appear to be directly phylogenetically related. KOSPep RNA3 showed the highest similarity to an Italian tomato variant (Y16926—91.985%) (Table S8), while KOSTom RNA3 exhibited the highest similarity (97.498%) to a Hungarian *Trifolium repens* variant (PQ462340) (Table S8).

### 2.3. Pepper Plants Were Infected with BBWV2 and PVY

**Figure 3:**
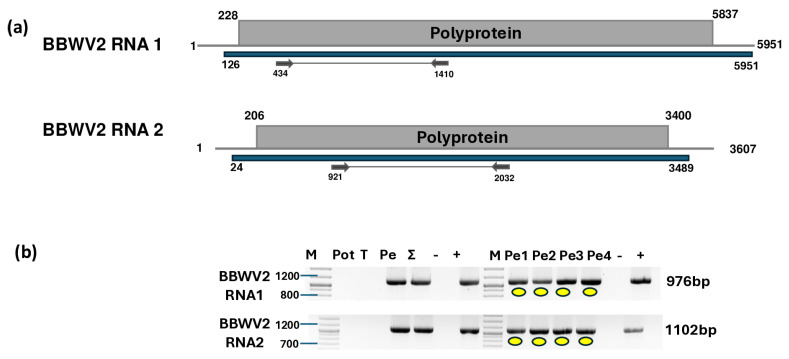
Results indicating the presence of BBWV2. (**a**) Cartoon representation of the BBWV2 genome and the positions of the primers used for RT-PCR validation. The blue line indicates the region covered by the HTS sequencing results. (**b**) Validation of the presence of BBWV2 using RT-PCR. M represents the GeneRuler 100 bp Plus DNA Ladder; Pot indicates potato; T denotes tomato; Pe indicates the pepper pool; Σ denotes the combined pool of Pot, T, and Pe that was sequenced; −/+ indicate negative and positive controls, respectively; and Pe1, Pe2, Pe3, and Pe4 denote individual pepper plants. Yellow dots indicate the products that were cloned and sequenced.

Seven and five contigs showed strong identity to BBWV2 RNA1 and RNA2, respectively. In total, 124,493 reads were mapped to RNA1, and 211,548 reads were mapped to RNA2, resulting in 96% and 99% coverage of the BBWV2 segments, respectively (Figure 3a, Table S4).

The validation of BBWV2 showed that only the pepper plants, including all four individuals, were infected with this virus. The PCR products originating from the individuals were cloned and Sanger-sequenced (GenBank accession number PV020942-48) (Table S6).

**Figure 4:**
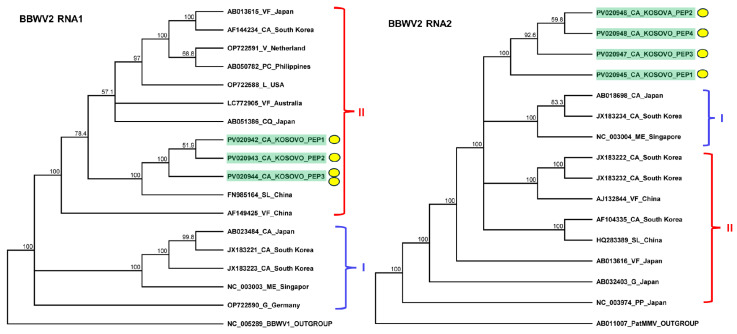
Phylogenetic analysis of the BBWV2 variants found in Kosovo. The analysis was conducted using Geneious Tree Builder with the Tamura–Nei model, the neighbor-joining method, and 1000 bootstrap replicates. Red and blue lines indicate clusters of BBWV2 subgroups. Yellow dots indicate the variants sequenced in this study. The sequences are indicated by their GenBank accession numbers. The middle panel of the legend indicates the host plant species using the following abbreviations: VF—*Vicia faba*, CA—*Capsicum annuum*, V—*Verbena*, PC—*Pogostemon cablin*, L—*Lactuca* spp., CQ—*Chenopodium quinoa*, LE—*Lycopersicon esculentum*, ME—*Megaskepasma erythrochlamys*, G—*Gentiana*, and PP—*Pogostemon patchouli*.

The sequencing of the amplified portions of RNA1 and RNA2 BBWV2 revealed three and four slightly different variants clustering together into subgroup II and between the two BBWV2 subgroups, respectively (Figure 4). These variants were significantly different from the BBWV2 variants in GenBank (78–92% identity for RNA1 and 80–86% identity for RNA2). The RNA1 of the BBWV2 KOSPep sequences was the most similar to a variant sequenced in China from a tomato (FN985164 in subgroup II with 92% identity), while RNA2 showed the closest similarity (AB013616 in subgroup I with 87%) to a variant sequenced from *Vicia faba* in Japan (Table S9).

**Figure 5:**
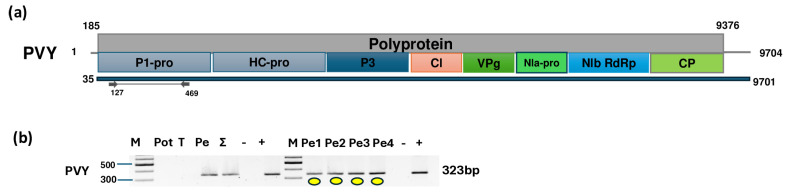
Results indicating the presence of PVY. (**a**) Cartoon representation of the PVY genome and the positions of the primers used for RT-PCR validation. The blue line indicates the region covered by the HTS sequencing results. (**b**) Results validating the presence of PVY RT-PCR. M represents the GeneRuler 100 bp Plus DNA Ladder; Pot indicates potato; T indicates tomato; Pe denotes the pepper pool; Σ denotes the combined pool of Pot, T, and Pe that was sequenced; −/+ denote negative and positive controls, respectively; and Pe1, Pe2, Pe3, and Pe4 denote individual pepper plants. Yellow dots indicate the products that were cloned and sequenced.

Six contigs and 610,666 reads mapped to PVY, covering 99% of the genome (Figure 5a, Table S4).

**Figure 6:**
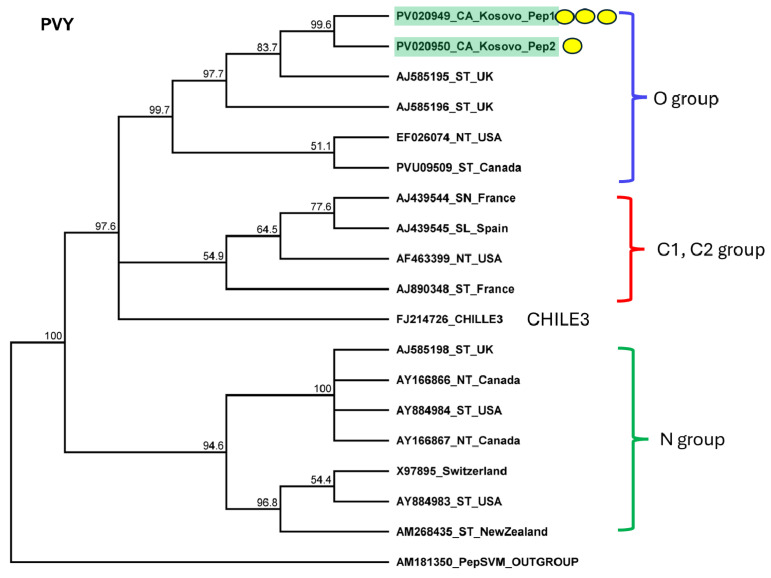
Phylogenetic analysis of the PVY variants found in Kosovo. The analysis was conducted using Geneious Tree Builder with the Tamura–Nei model, the neighbor-joining method, and 1000 bootstrap replicates. Green boxes highlight the pepper variants sequenced in this study. The colored line shows the subgroups of PVY. Yellow dots indicate the variants sequenced in this study. The sequences are indicated by their GenBank accession numbers. The middle panel of the legend indicates the host plant using the following abbreviations: ST—*Solanum tuberosum*, NT—*Nicotiana tabacum*, SN—*Solanum nigrum*, and SL—*Solanum lycopersicum*.

The validation of the virus’ presence confirmed that all sampled pepper individuals were infected, while potato and tomato were free of the virus. Sanger sequencing of the short, cloned portion of the virus showed very strong identity (above 96%) to the variants available in GenBank (Table S10). The KOSPep variants clustered together within the O clade (Figure 6).

### 2.4. All Sampled Pepper Plants Were Infected with PCV2 and BPEV

**Figure 7:**
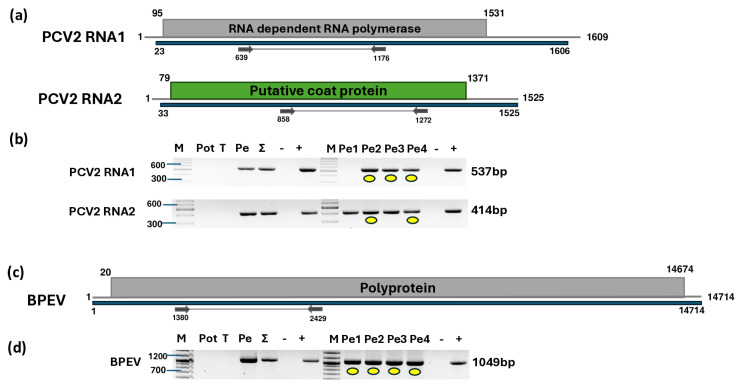
Results indicating the presence of PCV2 and BPEV. Cartoon representation of the (**a**) PCV2 and (**c**) BPEV genomes and the positions of the primers used for RT-PCR validation. The blue line indicates the region covered by the HTS sequencing results. (**b**) and (**d**) show the validation results indicating the presence of PCV2 and BPEV using RT-PCR, respectively. M represents the GeneRuler 100 bp Plus DNA Ladder; Pot indicates potato; T indicates tomato; Pe indicates the pepper pool; Σ denotes the combined pool of Pot, T, and Pe that was sequenced; −/+ represent negative and positive controls, respectively; and Pe1, Pe2, Pe3, and Pe4 denote individual pepper plants. Yellow dots indicate the products that were cloned and sequenced.

Two contigs were similar to PCV2 RNA1, and one contig could be mapped to the RNA2 of this virus. In total, 27,242 and 6786 reads mapped to RNA1 and RNA2, respectively, covering 99% of the entire viral genome (Figure 7a, Table S4). One BPEV-derived contig was built from the 54,178 mapped reads, covering 98% of the genome (Figure 7c, Table S4).

**Figure 8:**
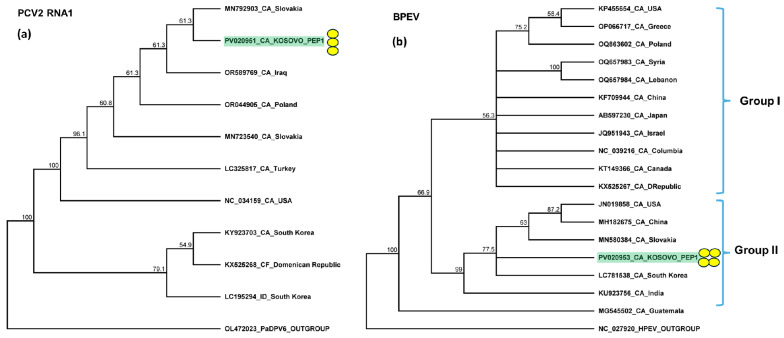
Phylogenetic analysis of the (**a**) PCV2 and (**b**) BPEV variants found in Kosovo. The analysis was conducted using Geneious Tree Builder with the Tamura–Nei model, the neighbor-joining method, and 1000 bootstrap replicates. Green boxes highlight the pepper variants sequenced in this study. Subgroups of BPEV are indicated. Yellow dots indicate the variants sequenced in this study. The sequences are indicated by their GenBank accession numbers. The middle panel of the legend presents the host plant species using the following abbreviations: CA—*Capsicum annuum*, CF—*Capsicum frutescens*, and ID—*Ixeridium dentatum*.

The validation of the presence of PCV2 and BPEV revealed that all of the pepper plants were infected with these latent viruses. However, the amplification of RNA1 of PVC2 in the first pepper plant yielded no product, which could be explained by a slight variation at the primer annealing site of the PVC2 variant present in this individual. The sequencing of the amplified part of PCV2 showed that the sequences of the products originating from RNA2 were identical or highly similar (greater than 99% identity) to other PCV2 RNA2 sequences available in GenBank (Table S11). The RNA1 sequences of the variants found in Kosovo were identical and slightly more variable than those of RNA2, sharing over 97% identity with other PCV2 variants (Table S11). The closest homolog of the KOSPep variant was a variant sequenced in Slovakia (Figure 8a).

The sequencing of the amplified portion of BPEV KOSPep showed that the sequences of the variants were identical and clustered to group II of this virus (Figure 8b). This sequence was 88–99% identical to the sequences of the BPEV variants available from GenBank (Table S12).

### 2.5. Pepper Plants Were Infected with Ranunculus White Mottle Virus

**Figure 9:**
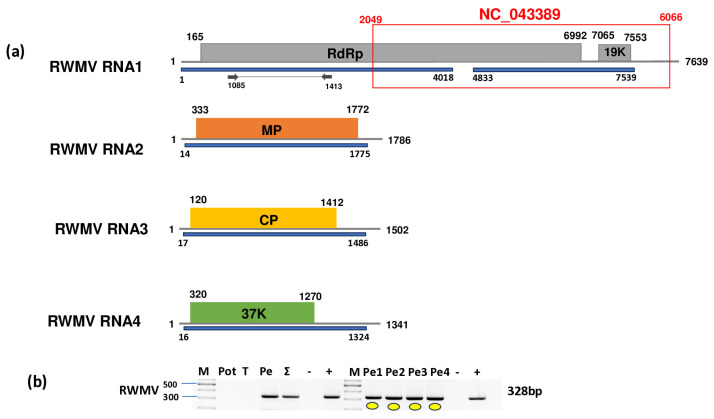
Results indicating the presence of RWMV. Cartoon representation of the RWMV genome. (**a**) RNA1, RNA2, RNA3, and RNA4 and the positions of the primers used for RT-PCR validation. The blue line indicates the region covered by the HTS sequencing results. The red box shows the region of the GenBank reference genome of the virus. (**b**) Validation of the presence of RWMV using RT-PCR. M represents the GeneRuler 100 bp Plus DNA Ladder; Pot indicates potato; T indicates tomato; Pe indicates the pepper pool; Σ denotes the combined pool of Pot, T, and Pe that was sequenced; −/+ denote negative and positive controls, respectively; Pe1, Pe2, Pe3, and Pe4 denote individual pepper plants. Yellow dots indicate the products that were cloned and sequenced.

We identified two contigs that were highly similar to RWMV RNA1. The mapping of the reads revealed 227 mapped reads, covering 99% of the reference genome. A direct BLAST search of these contigs revealed hits to an RWMV RNA1 (OR879093) sequence that was 3621 nt longer than the NC_043389 reference genome. OR879093 was sequenced at DSMZ, together with three other RNAs originating from different segments of RWMV. BLAST searches of the contigs against these four RWMV genome segments revealed additional hits: three contigs mapped to RNA1, while an additional three were highly similar to RNA2, RNA3, and RNA4 of the virus. The remapping of the reads to every four segments resulted in 494, 1037, 2460, and 8800 mapped reads to RNA1, RNA2, RNA3, and RNA4, covering 98% of the viral genome for RNA1, 98% for RNA2, 97% for RNA3, and 97% for RNA4 (Figure 9), (Table S4).

**Figure 10:**
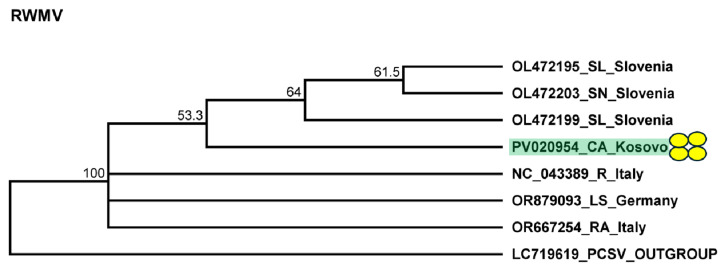
Phylogenetic analysis of the RWMV variant found in Kosovo. The analysis was conducted using Geneious Tree Builder with the Tamura–Nei model, the neighbor-joining method, and 1000 bootstrap replicates. Green boxes highlight the pepper variants sequenced in this study. Yellow dots indicate the variants sequenced in this study. The sequences are indicated by their GenBank accession numbers. The middle panel of the legend presents the host plant using the following abbreviations: SL—*Solanum lycopersicum*, SN—*Solanum nigrum*, CA—*Capsicum annuum*, R—*Ranunculus* spp., LS—*Lactuca sativa*, and RA—*Ranunculus asiaticus*.

To validate the presence of the virus, we assessed the presence of RNA1. Our results showed that all of the pepper individuals were infected with RWMV. The sequences of the cloned portions of the RMWV variants present in the individuals were 92–95% identical to the other variants of the virus with sequences available in GenBank (Table S13). The variant present in Kosovo clustered with the variants sequenced in Slovenia (Figure 10).

## 3. Discussion

Virome determination in crops has been revolutionized in the past decade. The usage of HTS-based metagenomic studies as surveys has become routine, making it possible to fully map the presence of viruses in different crops at distinct geographical locations. This study represents the first investigation into plant viruses affecting potato, tomato, and pepper varieties in Kosovo using nucleic acid-based molecular methods. Previously, no such study had been conducted in the country, particularly on these economically significant crops. Although we tested only a few plants and RNA sequencing was performed using one unified pool, we were able to identify the presence of six viruses previously not identified in Kosovo. The presence of these viruses was clear, with a large number of viral reads evenly distributed across the viral genome, confirming the robustness of this technique. For HTS diagnostics, RNA-seq libraries were prepared from pools containing a mixture of individual plant extracts. To validate the HTS results, RT-PCR was employed as an independent confirmation method, ensuring reliability and allowing us to identify the infected plant species and individuals. The application of HTS allowed for unbiased and efficient virus detection, highlighting its significance as a powerful tool for plant virus diagnostics. As a sensitive diagnostic tool, this type of pooling strategy is commonly used when RNAseq is employed. The advantage of pooling is that it allows for the simultaneous monitoring of the viromes of multiple plants. The limitation of the technique is that, if only some plants are infected, the RNA originating from the other plants can dilute the sample, even to levels below the detection limit; consequently, infection with viruses at low concentrations can go unnoticed. In our case, an investigation of the RNA originating from 12 plants detected infection with six viruses. However, while it is highly unlikely, we cannot exclude the possibility that infection with more viruses at low concentrations could have occurred.

Although we sampled the potato plants based on their virus-like symptoms, they were found to be free from viruses and viroids. Of the four tomatoes that were tested based on their virus-like symptoms, only one was found to be infected by one virus, namely CMV. Of note, AMV, TSWV, ToMV, and TMV infections were not detected in the samples from Kosovo. In contrast to this low virus infection rate of potatoes and tomatoes, all four pepper plants were infected by six viruses. Since its identification in 1916, CMV has been detected in over 1200 plant species across more than 100 host plant families, establishing itself as a significant pathogen in temperate regions and increasingly in tropical areas [13,44]. CMV exhibits considerable genetic diversity, with strains categorized into three subgroups (IA, IB, II), showing 70–98% nucleotide identity [13]. While single infections were generally the most common, double infections were the primary type of mixed infection observed [3]. In some cases, severe symptoms such as leaf malformation and fruit deformities can occur, especially in the context of mixed infections with other viruses like TMV or PVY [1,45,46]. In our case, we found CMV infection in all peppers and one tomato plant. The phylogenetic analysis of the partial RNA3 sequences of the variants showed that, while the KOSTo variant found in the tomato plant belonged to the less symptomatic clade II, the pepper-infecting KOSPep variant clustered in clade IB. For this KosPep variant, we partially amplified and sequenced RNA1 and RNA2. The phylogenetic analysis of these segments of the virus showed that RNA1 clustered to clade IB, similarly to RNA3. However, the partial RNA2 sequence clustered with clade IIA. This difference in the clustering of the segments indicated the potential reassortant nature of the KOSPep variant. A RT-PCR-RFLP study investigating the CMV subgroup in various host plants revealed another sample from Serbia with a dominant IA subgroup origin. In that study, neither CMV of the IB clade nor the presence of any reassortant was detected [17]. According to the EPPO website (https://gd.eppo.int/taxon/CMV000/distribution (accessed on 28 February 2025), CMV is widespread in the Balkan area. This notion is also supported by the long list of CMV sequences deposited in NCBI GenBank that originated from Balkan countries and were sequenced from a wide range of plant hosts (Table S1). Unfortunately, these sequences are almost exclusively partial sequences of RNA3, so it is not possible determine the frequency of the appearance of a reassortant CMV and its possible origin. BBWV isolates are divided into two species: BBWV1 and BBWV2. Although these species exhibit analogous genome structures and functionalities, their nucleotide sequence identity remains comparatively low, fluctuating between 39% and 67%. This disparity is largely attributable to negative selection and recombination, which serve as pivotal mechanisms fostering this diversity [4,23]. BBWV2 is capable of co-infecting plants along with other viruses, including CMV, PMV, PMMV, and PVY, and such co-infections often exacerbate the severity of diseases affecting crops [24]. All of the sampled pepper plants were infected with BBWV2. The phylogeny of BBWV2 variants showed the presence of two clades (I and II). KOSPep isolates clustered together, suggesting their on-site origin. However, the phylogeny of their RNA1 and RNA2 segments showed that, while RNA1 clustered into clade II, RNA2 clustered separately from the originally described clades. This finding suggests the reassortant or separate origin of these KOSPep variants. PVY causes symptoms such as stunting, leaf distortion, vein clearing, necrosis, and mosaic patterns. PVY is globally distributed and has a significant economic impact [25,27]. PVY is mainly distributed by infected planting material and mechanical distribution, and its main host is potato. The potatoes sampled in Kosovo did not harbor this virus. Unfortunately, this was not the case for pepper, as all of the sampled individuals were infected with this virus. The KOSPep PVY variant clustered to clade O. PVY was previously described in the Balkans, and there are several PVY sequences originating from Balkan countries deposited in GenBank (Table S1). The sequences of PVY variants infecting pepper are available at NCBI GenBank; those from Bosnia and Herzegovina, Croatia, Macedonia, and Serbia have been reported [47], suggesting that pepper is frequently infected with this virus. Mixed infections involving PCV2 and other cryptoviruses or host-specific pathogenic viruses are common in pepper plants [30]. In Europe, PCV2 was first reported in Slovakia after testing various symptomatic sweet pepper and chili plants in 2018 and 2019 [37]. The observed symptoms, such as mosaic patterns and leaf mottling, could not be definitively attributed to PCV2 as the plants were also infected with other viruses. PCV2 has also been reported in Poland, where it is widespread and has been found in co-infection with other viruses [12]. A phylogenetic analysis conducted on PCV2 revealed three distinct groups of isolates based on the RNA1 and RNA2 sequences [9]. Overall, the variability in the symptoms associated with PCV2 infection underscores the need for ongoing surveillance and research to fully understand its impact on pepper crops. PCV2 was detected in all of the sampled peppers. The KOSPep variant of PVC2 had the closest similarity to the Slovak isolate; however, the exclusively high similarity of the PCV2 RNA2 to other isolates (99–100%) did not allow us to obtain detailed information about its phylogeny. Similarly to PCV2, BPEV is very often found infecting pepper. BPEV is a persistent virus infecting pepper plants exclusively through seeds [33]. Although it can cause mild crinkling and chlorosis in bell peppers, in *Capsicum* sp., its presence often remains latent, with variable effects on the fruit yield and quality [33,34]. The symptom severity differs between cultivars, with hot peppers showing milder effects [9]. All of the pepper plants contained this virus, and this KOSPep variant clustered with Group II of the virus. We found a full genome for all four RNAs of RWMV, whose reference genome was only a partial sequence of RNA1. The full genome (OR879093-6) was sequenced at the DSMZ from a *Lactuca sativa* plant originating from Bodensee, Germany (GenBank record only). RWMV was originally characterized as a virus with three RNA segments [43,48]; however, recent reports, including our work using RNAseq for virome determination, have confirmed the presence of RNA4 [42]. It is very difficult to connect RWMV’s presence to significant crop losses, as it is typically present in mixed infections. Although its vector has not yet been identified, it is possible that, similarly to other ophioviruses, root-infecting *Olphdidium* species act as its vectors. The presence of RWMV results in symptoms that can be confused with those of the damaging TSWV, emphasizing the need to develop sensitive diagnostic tools to prevent misdiagnosis and the use of inappropriate management [6,40,41].

The precise route through which the detected plant viruses were introduced into Kosovo remains uncertain. However, it is plausible that these viruses were transmitted via multiple pathways, including the import of infected planting material, such as seeds, seedlings, or vegetative propagules, from neighboring countries or other regions. The informal movement of plant materials through local markets or across borders and the informal exchange of plant material among local growers, without stringent phytosanitary controls, could easily contribute to the introduction and spread of these viruses. PCV2 and BPEV have been always detected in pepper virome surveys when HTS has been used as a diagnostic method. This suggests that they are highly associated with this species, while the other viruses can spread through insect vectors.

Although specific data on local virus vectors in Kosovo are limited, aphids, whiteflies, thrips, and leafhoppers are present in Kosovo’s agroecosystems. Future studies focusing on the identification and monitoring of these vector species and their population dynamics in Kosovo would help to understand virus epidemiology and are essential in implementing effective management strategies.

Insect vectors can multiply on the weeds and wild plants surrounding the crop fields, and these vector hosts can act as virus reservoirs. It is possible that the identified viruses could have been present in Kosovo for a long time and spread to the crops through the activity of the vectors. However, to answer this question, further studies on virome identification in weeds and wild plants in Kosovo are needed.

## 4. Materials and Methods

### 4.1. Sample Collection and Preparation

In the summer of 2023, leaf samples of potatoes, tomatoes, and pepper plants showing virus-like symptoms were collected from various regions across Kosovo, including Pestove, Barilevë, Bradash, and Gjurakoc (Table S2). In total, 12 plant samples were used for virome determination. Briefly, 5 µL total nucleic acid extracted from the four samples was combined to generate three 20 µL species-specific pools. From these pools, 5 µL was combined into a single pool containing RNA from all three species. The potato cultivar *Agria* was sampled at Pestove and Barilevë. The traditional tomato cultivar *Shijaka* was sampled at Bradash and Gjurakoc, while the cultivar *Belle* was sampled from Barilevë. Pepper was sampled from Barilevë and Bradash. At Barilevë, *Somborka* pepper cultivars were grown from commercially purchased seeds after tomato was grown as the previous crop in the rotation. Weed management was achieved using herbicides, resulting in 3% weed presence. At Bradash, *Shorok Shari*, locally known as the “Babure” cultivar, was grown in open fields and propagated as seedlings prepared using the traditional Albanian method with a suitable substrate for quality seedling production. In the previous year, tomato was also the previous crop, and herbicide application reduced weed presence to 5%.

### 4.2. Sampling and Nucleic Acid Extraction

The leaves of symptomatic plants were collected, placed in calcium chloride (CaCl_2_) to preserve the samples’ integrity, and transported to Hungary for further analysis. The total nucleic acids were extracted from the collected leaves using the phenol–chloroform method [49] for potato and tomato, while TRIzol (Sigma Alrich, Merck KGaA, Darmstadt, Germany) was used for the peppers. Aliquots of the total nucleic extracts were pooled according to the individuals and by location to represent the viral content within each field. Subsequently, the generated pools were combined into a single tube for comprehensive analysis. Retaining the individual nucleic acid extracts alongside this pooling approach enabled the detection of any virus present in any of the sampled plants.

### 4.3. High-Throughput Sequencing

For HTS, the total nucleic acid pool was DNase-treated using Thermo Scientific DNase I, (Thermo Scientific, Waltham, MA, USA), RNase-free, following the manufacturer’s recommendation. The DNase-treated sample, containing high-quality pure RNA, was sent for ribodepleted RNA sequencing, which was performed as 150 bp paired-end ncRNA sequencing on the Illumina platform from NOVOGEN as a service. The sequenced reads were deposited in NCBI GenBank (SRA:PRJNA1215027).

### 4.4. Bioinformatics Analysis of the HTS Results

The fastq files of the sequencing were analyzed using the CLC Genomic Workbench of Qiagen (version number, 20.0.4, Qiagen, Hilden, Germany). Following data import, we conducted trimming and generated FastQC reports. Subsequently, we prepared contigs from the paired reads (de novo assembly) (see Table S3 for the initial statistics). A BLAST search of contigs to the reference genomes of the currently known plant-infecting viruses (downloaded from NCBI GenBank 31_07_2023) revealed the list of viruses present in the sampled plants. The reads were also directly mapped to the reference genomes of viruses for which contigs with a zero E value were found. Consensus sequences prepared from this mapping, as well as alignment, allowed us to calculate the coverage of the viral genome by virus-derived sequences (Table S4).

### 4.5. Validation of HTS Using RT-PCR

RT-PCR was conducted to validate the results of the bioinformatics analysis.

RNA pools prepared from the total nucleic acid extracts of potato, tomato, and pepper and the individual tomato and pepper plants were used as templates for cDNA synthesis. cDNA synthesis was performed using the RevertAid First Strand cDNA Synthesis Kit or Thermo Scientific Maxima™ Reverse Transcriptase (Thermo Fisher Scientific, Waltham, MA, USA) with random primers, following the manufacturer’s instructions.

The cDNA quality was confirmed in an RT-PCR reaction using actin-specific primers. Virus-specific primers were utilized to amplify various genomic regions of the virus of interest. The primers used for the amplification were published or designed based on the contig sequences (Table S5). RT-PCR was conducted with Q5 Hot Start High-Fidelity DNA Polymerase (New England Biolabs, Ipswich, MA, USA). The optimal annealing temperature of the primers was determined experimentally and is included in Table S5. Viral-specific PCR products were purified using the NucleoSpin Gel & PCR Clean-Up Kit (Macherey and Nagel), cloned into GeneJET vectors (Thermo Fisher Scientific), and Sanger-sequenced, which was performed as a service.

Sequences were deposited into GenBank (PV020939-PV020954 and PV102684). Sequence variants with the same sequence were deposited only once. A detailed list of the occurrence of the variants is provided in Table S6.

### 4.6. Phylogenetic Analysis of the Detected Viral Strains

To compare and phylogenetically analyze the virus variants present in the samples, multiple sequence alignments were performed using Geneious Prime (version number: 2024.0.7), with the MUSCLE algorithm. The key parameters of the variants used for the phylogeny are summarized in Table S7. Evolutionary relationships were inferred using the Jukes–Cantor model and the neighbor-joining method. Phylogenetic trees were constructed based on the optimal model for each alignment, with 1000 bootstrap replicates to assess reliability. The trees were scaled, with the branch lengths representing the number of substitutions per site. The closest relatives of the viruses, as indicated in the tree legend, were used as outgroups. The phylogenetic analysis was conducted using the sequenced amplified parts of the detected viruses: for CMV, 1194 nt partial RNA1, 1759 nt partial RNA2, and 1351 nt partial RNA3; for BBWV2, 976 nt partial RNA1 and 1102 nt partial RNA2; for PVY, 323 nt partial P1-pro; for PCV2, 537 nt partial RNA1 and 414 nt partial RNA2; for BPEV, 1049 nt partial polyprotein coding sequence; and for RWMV, 328 nt partial RNA1.

## 5. Conclusions

In our pilot study, we found multiple infections with six viruses by sampling only four individual pepper samples at two locations in Kosovo. The high infection rate of the pepper plant with multiple viruses was an unexpected result. While the presence of PCV2 and BPEV appeared to be latent and universal in pepper, at present, there is a lack of documented evidence regarding the potential effects and symptomatology of RWMV infection in this plant species. Thus, further investigation is warranted to elucidate this notion and advance our understanding. Infection with CMV, BBWV2, and PVY can affect plants’ physiology and reduce the quality and quantity of the harvested product, indicating that infection should be avoided during propagation and planting. The introduction of cheap and reliable diagnostics and appropriate phytosanitary regulations could ensure safe and high-quality crop production in the future. Overall, these findings underscore the importance of adopting advanced molecular techniques such as HTS for plant virus surveillance and management. This study establishes a foundation for future epidemiological research, aiding in the development of strategies to mitigate the impact of plant viruses in the region.

## Data Availability

FASTQ files of the sequenced libraries were deposited in the NCBI as an SRA and can be accessed through series accession number PRJNA1215027. Partial viral sequences of the viral were deposited into GenBank (PV020939-PV020954 and PV102684).

## Supplementary Materials

The following supporting information can be downloaded at: https://www.mdpi.com/article/10.3390/plants14091273/s1, Table S1: List of GenBank records of CMV, BBWV2, PVY, PCV2, BPEV, and RWMV from Balkan countries; Table S2: Basic information of the sampled plant species and individuals; Table S3: Initial RNAseq statistics; Table S4: Summary of the bioinformatics analysis; Table S5: Sequences of the PCR primers used for virus detection; Table S6: Summary of the virus sequences deposited into NCBI GenBank; Table S7: Virus sequences used for phylogenetic analysis; Table S8: Percent identity matrix of the CMV sequences that were considered in the phylogenetic analysis; Table S9: Percent identity matrix of the BBWV2 sequences that were considered in the phylogenetic analysis; Table S10: Percent identity matrix of the PVY sequences that were considered in the phylogenetic analysis; Table S11: Percent identity matrix of the PCV2 sequences that were considered in the phylogenetic analysis; Table S12: Percent identity matrix of the BPEV sequences that were considered in the phylogenetic analysis, Table S13: Percent identity matrix of the RWMV sequences that were considered in the phylogenetic analysis.
